# Machine-learning-based models to predict cardiovascular risk using oculomics and clinic variables in KNHANES

**DOI:** 10.1186/s13040-024-00363-3

**Published:** 2024-04-22

**Authors:** Yuqi Zhang, Sijin Li, Weijie Wu, Yanqing Zhao, Jintao Han, Chao Tong, Niansang Luo, Kun Zhang

**Affiliations:** 1 School of Computer Science & Engineering, Beihang University, Beijing, China; 2 Department of Cardiology, the Eighth Affiliated Hospital, Sun Yat-sen University, Shenzhen, China; 3 State Key Laboratory of Virtual Reality Technology and Systems, Beihang University, Beijing, China; 4 Department of Interventional Radiology & Vascular Surgery, Peking University Third Hospital, Beijing, China; 5 Department of Cardiology, Sun Yat-sen Memorial Hospital, Sun Yat-sen University, Guangzhou, China; 6 Department of Cardiology, The Seventh Affiliated Hospital of Sun Yat-sen University, Shenzhen, China

**Keywords:** Triglyceride-glucose index, Atherogenic index of plasma, Oculomics, Cardiovascular risk, Machine learning

## Abstract

**Background:**

Recent researches have found a strong correlation between the triglyceride-glucose (TyG) index or the atherogenic index of plasma (AIP) and cardiovascular disease (CVD) risk. However, there is a lack of research on non-invasive and rapid prediction of cardiovascular risk. We aimed to develop and validate a machine-learning model for predicting cardiovascular risk based on variables encompassing clinical questionnaires and oculomics.

**Methods:**

We collected data from the Korean National Health and Nutrition Examination Survey (KNHANES). The training dataset (80% from the year 2008 to 2011 KNHANES) was used for machine learning model development, with internal validation using the remaining 20%. An external validation dataset from the year 2012 assessed the model’s predictive capacity for TyG-index or AIP in new cases. We included 32122 participants in the final dataset. Machine learning models used 25 algorithms were trained on oculomics measurements and clinical questionnaires to predict the range of TyG-index and AIP. The area under the receiver operating characteristic curve (AUC), accuracy, precision, recall, and F1 score were used to evaluate the performance of our machine learning models.

**Results:**

Based on large-scale cohort studies, we determined TyG-index cut-off points at 8.0, 8.75 (upper one-third values), 8.93 (upper one-fourth values), and AIP cut-offs at 0.318, 0.34. Values surpassing these thresholds indicated elevated cardiovascular risk. The best-performing algorithm revealed TyG-index cut-offs at 8.0, 8.75, and 8.93 with internal validation AUCs of 0.812, 0.873, and 0.911, respectively. External validation AUCs were 0.809, 0.863, and 0.901. For AIP at 0.34, internal and external validation achieved similar AUCs of 0.849 and 0.842. Slightly lower performance was seen for the 0.318 cut-off, with AUCs of 0.844 and 0.836. Significant gender-based variations were noted for TyG-index at 8 (male AUC=0.832, female AUC=0.790) and 8.75 (male AUC=0.874, female AUC=0.862) and AIP at 0.318 (male AUC=0.853, female AUC=0.825) and 0.34 (male AUC=0.858, female AUC=0.831). Gender similarity in AUC (male AUC=0.907 versus female AUC=0.906) was observed only when the TyG-index cut-off point equals 8.93.

**Conclusion:**

We have established a simple and effective non-invasive machine learning model that has good clinical value for predicting cardiovascular risk in the general population.

**Supplementary Information:**

The online version contains supplementary material available at 10.1186/s13040-024-00363-3.

## Introduction

### Cardiovascular disease and oculomics

Cardiovascular disease (CVD) is a profound global public health challenge and ranks among the primary contributors to the worldwide disease burden [1, 2]. CVD remains a leading cause of both mortality and morbidity on a global scale, taking an estimated 17.9 million lives each year, even with advancements in preventive strategies and therapeutic techniques. The assessment of cardiovascular risk assumes paramount importance in global public health.

Patients with CVD are more easily found to have metabolic abnormalities, such as insulin resistance, hyperglycemia, and dyslipidemia [3, 4]. Recently, some new indicators calculated through blood glucose and serum lipids, such as the triglyceride-glucose index (TyG-index) and the atherogenic index of plasma (AIP) demonstrated to be a higher correlation with CVD [5]. Furthermore, TyG-index and AIP are good indicators for cardiovascular risk [5, 6]. Different cut-off point values for TyG-index and AIP can reflect the cardiovascular risks of different groups of people.

**Fig. 1:**
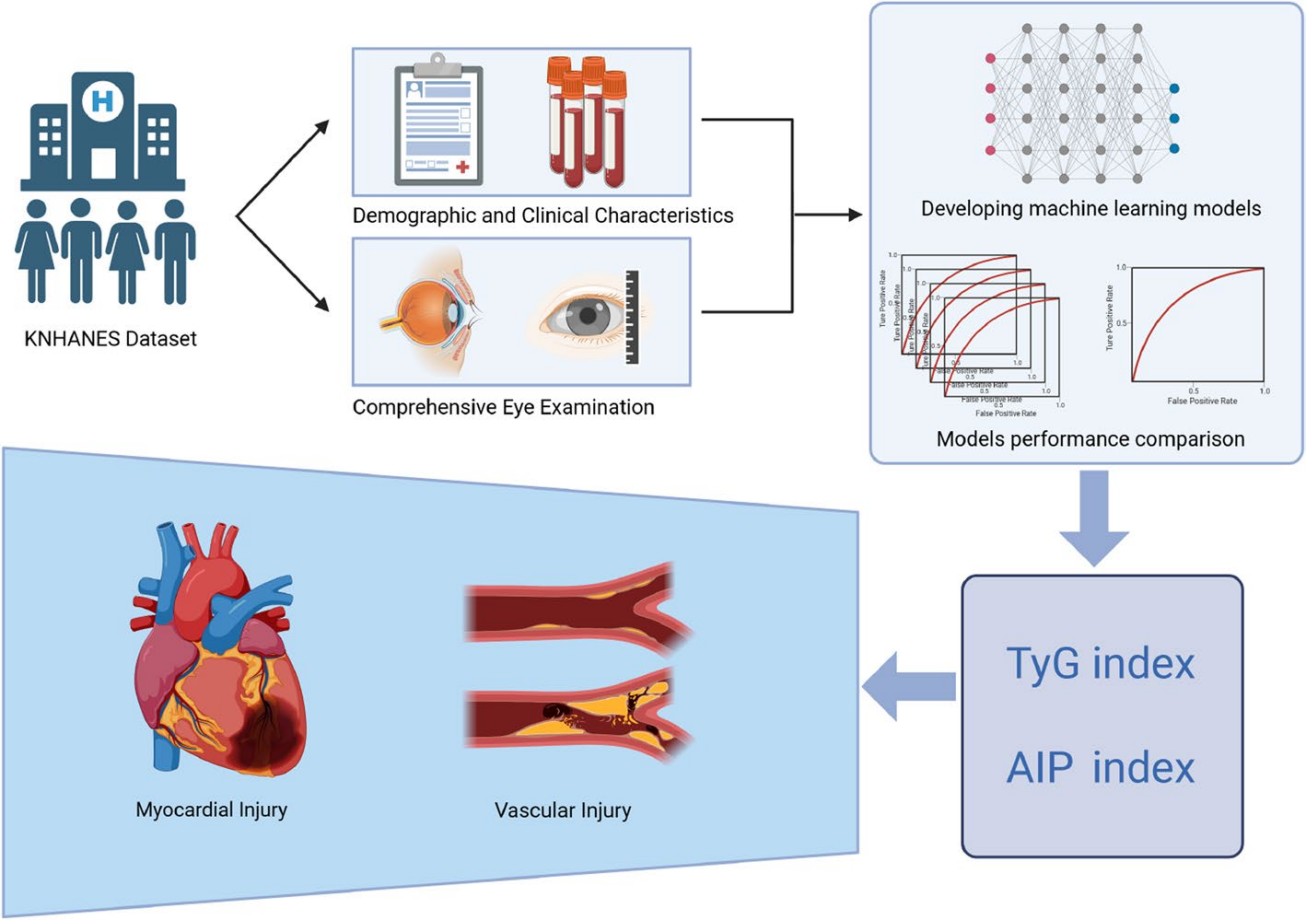
Central illusion. Machine-learning-based cardiovascular risk prediction using data extracted from oculomics and clinic variables in Korean National Health and Nutrition Examination Survey (KNHANES)

Previous studies have confirmed that ophthalmology diseases are closely related to CVD. Eye is an organ that can directly reflect microvascular changes [7]. The pathogenesis of many CVDs also leads to ocular changes [8]. Therefore, it is feasible to use the pathology of eyes to predict CVD. Oculomics is a newly proposed concept in recent years [9]. It refers to the blending of big data, artificial intelligence (AI), and ocular imaging to identify retinal biomarkers of systemic disease [10, 11]. AI has been extensively employed in the medical field for several years, automatically uncovering intrinsic patterns and connections between data variables and related diseases [12–15]. In the present study, we leveraged data from the Korean National Health and Nutrition Examination Survey (KNHANES) to develop and validate a machine-learning model for predicting cardiovascular risk based on variables encompassing clinical questionnaires and oculomics, as shown in Fig. 1. This model effectively anticipates the range of TyG-index and AIP, which reflect the level of cardiovascular risks.

## Materials and methods

### Dataset

This study used a comprehensive health examination dataset based on the Korean National Health and Nutrition Examination Survey (KNHANES IV and V; available online at https://knhanes.kdca.go.kr/knhanes/eng) conducted from the year 2008 to 2012. The KNHANES surveyed both comprehensive ophthalmologic examinations and DEXA only during this period. The study protocol was approved by the Institutional Review Board of the Korean Center for Disease Control and Prevention (No. 2008-04EXP-01-C, 2009-01CON-03-C, 2010-02CON-21-C, 2011-02CON-06-C, and 2012-01EXP-01-2C), and data collection was approved by the Institutional Review Board of the Korean National Institute for Bioethics Policy. All participants signed consent forms for the use of their health information for data collection of the KNHANES. The KNHANES is a nationwide, population-based, cross-sectional survey conducted by the Division of Chronic Disease Surveillance of the Korea Centers for Disease Control and Prevention [16]. This project randomly selected all participants from 200 (2008-2009) and 192 (2010-2011) enumeration districts using stratified sampling in which the following factors were considered: population, sex, age, regional area, and type of residential area. The KNHANES comprises health records based on health interviews, health examinations, and nutrition surveys. Each participant completed a questionnaire containing information such as age, household income, alcohol use, smoking status, hypertension, and diabetes [17].

**Fig. 2:**
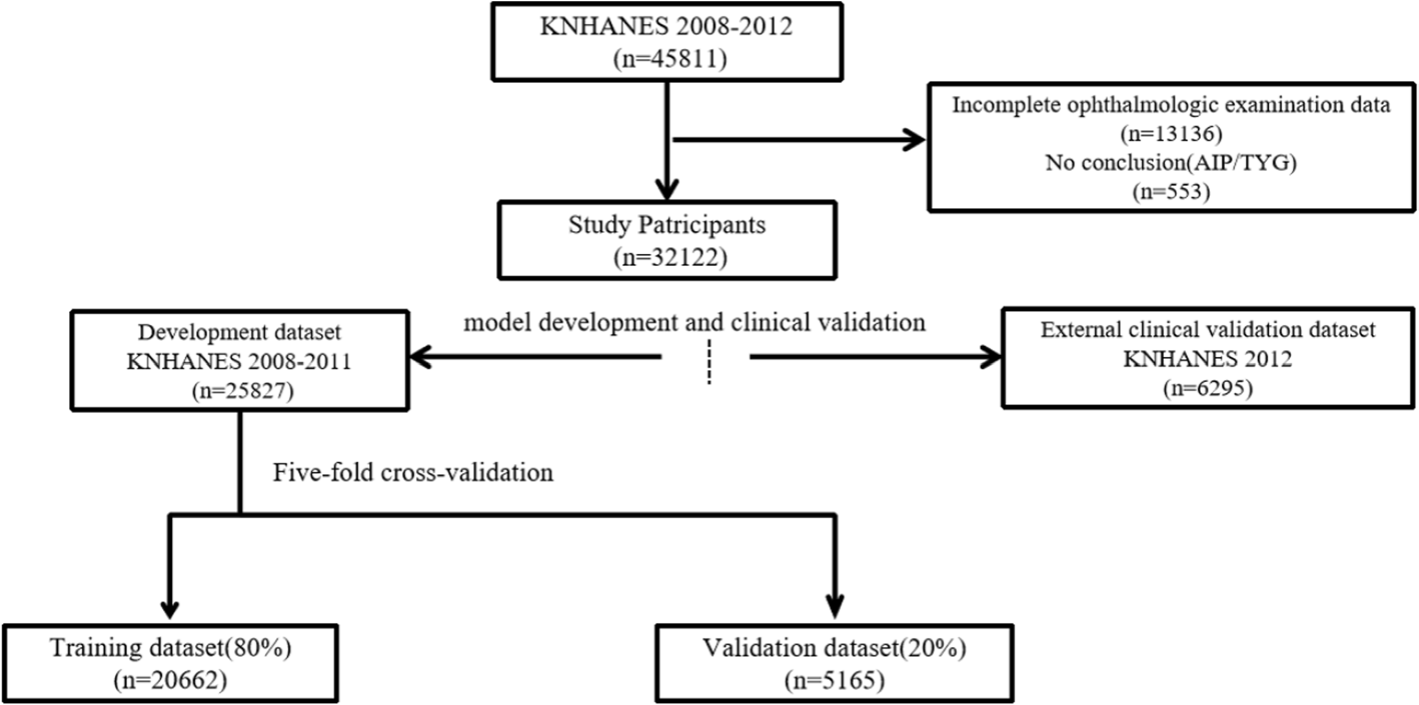
Schematic depiction of the data screening flow of the KNHANES from 2008 to 2012. Arrows indicate the data screening process. And the experimental process is described in the “Results” section. The details about the specific process of our experiment are shown in Supplementary Fig. S3

We conducted data curation based on the KNHANES dataset. Initially, individuals lacking either clinical variables or oculomics variables (*N*=13136), as well as those with missing TyG-index or AIP values (*N*=553), were excluded. Following this filtration process, a cohort of 32122 participants was included in this study. Then we utilized the KNHANES data from year 2008 to 2011 for model development and internal validation, exploring and verifying the associations between input variables and TyG-index as well as AIP. The model construction employed a five-fold cross-validation method. The training set, comprising 80% of randomly selected data from the year 2008 to 2011, was employed for machine learning model development, as the internal validation set encompassed the last 20% of randomly chosen data from this database. And the external validation set, sourced from the year 2012, was used to evaluate the model’s ability to predict AIP and TyG-index for previously unseen cases (Fig. 2). The code of this research is open source and can be accessed at “https://github.com/RickyZhang901/ML-Based-AIP_TYG”.

#### Key variables definition

The TyG-index was calculated as ln [TG (triglycerides) (mg/dL) $\times$ FBG (fasting blood glucose) (mg/dL)/2], derived from previous studies [18, 19]. The AIP is a logarithmically transformed ratio of TG to HDL-C (High density lipoprotein cholesterol) in molar concentration (mmol/L), and it is mathematically derived from log (TG/HDL-C) [20]. Regarding the outcomes, we selected different cut-off points for TyG-index and AIP. Their different tangent values can reflect the cardiovascular risk of different groups of people. For the TyG-index endpoint, we chose 8.0 [21], the upper one-third value of TyG values (8.75) [22], and the upper one-fourth value of TyG values (8.93) [23]. As for the AIP endpoint, 0.318 [20] and 0.34 [24] were chosen as the cut-off points. Endpoint values beyond these numerical cut-off points were deemed to indicate high cardiovascular risk, while values below were considered low risk.

#### Ophthalmic examination

In this study, we analyzed oculomics measurements graded by ophthalmologists. A comprehensive eye examination was conducted by the Korean Ophthalmological Society (KOS) using a vehicle equipped with ophthalmic devices. Trained ophthalmologists measured the eyelid positions of all participants. Marginal reflex distance 1 (MRD1) was defined as the distance from the upper eyelid margin to the corneal light reflex in the primary position (Supplementary Table S1). MRD1 values were obtained, and blepharoptosis was defined as an MRD1 of less than 2mm for either eye [25]. Ophthalmologists made a differential diagnosis of blepharoptosis with particular attention to avoiding misdiagnosis of pseudoptosis and dermatochalasis. The levator muscle function test was also performed by measuring the upper eyelid excursion from downgaze to upgaze (Supplementary Table S1), excluding any influence of frontalis muscle function, and sorted into normal ($\ge$12mm) and decreased levator function ($\le$11mm). Standardized slit-lamp examinations were performed to diagnose pterygium and cataracts. Fundus photographs were obtained using a digital fundus camera (TRC-NW6S; Topcon, Tokyo, Japan). For each eye of each participant, a 45^∘^ digital retinal fundus image centered on the macula and fovea was obtained. All fundus photographs were subjected to preliminary and detailed grading. Preliminary grades for retinal diseases and optic discs were assigned to the retinal images by trained ophthalmologists, and multiple retinal specialists performed detailed grading. After grading the fundus photographs, glaucoma was defined according to a previous study [26]. When the conditions of the two eyes from one participant differed, the worse eye was chosen for the analysis. The KOS National Epidemiologic Survey Committee periodically trained ophthalmologists to control the quality of the survey.

### Statistical analysis

#### Population baseline table

Statistical analyses were performed with SPSS version 24.0 (IBM Corp, Armonk, NY, USA), Python 3, and R version 4.2.2 (www.R-project.org). Continuous variables were described as mean ± standard deviation (SD) and compared by independent T-test or ANOVA. Categorical variables were described as numbers (percentages) and compared using chi-square tests.

#### Processing missing value

In this study, we employed polynomial interpolation to handle missing values in the dataset. Polynomial interpolation, a commonly utilized technique, fills in the missing values by constructing curves between known data points, preserving the overall trend of the data more effectively without introducing excessive noise. Thus, this method maintains the relative smoothness and continuity of the data. We deleted those variables with more than 30% missing and processed the remaining variables with missing value filling only in the internal dataset, which from the year 2008 to 2011 KNHANES.

#### Spearman correlation analysis

Within this research, we utilized Spearman correlation analysis to identify variables strongly correlated with the target variable. Spearman correlation analysis, a nonparametric statistical method, measures the monotonic relationship between two variables, which is particularly suitable for data that does not adhere to the assumption of a linear relationship. And the *p*<0.001 is considered as a statistically significant difference. Through Spearman correlation analysis, we effectively reduced the dimensions of the dataset, thereby enhancing modeling efficiency.

#### Features construction

In addition to the selected features, we employed AutoFeat [27] for feature construction. This automated feature engineering tool facilitates extracting more insightful features from the raw data through a sequence of feature engineering steps, encompassing both directly derived features and interaction features. This augmentation would furnish our model with a more extensive wellspring of information, contributing to enhancing the model’s generalizability and predictive accuracy. From the processing missing value step to the feature construction step, we only used the data set from 2008 to 2011 of KNHANES to avoid going against established data science best practices. Supplementary Fig. S3 details the process of building our model and the different uses of data sets from different years.

#### Metrics for evaluation

To evaluate the performance of the machine learning models, we computed the Area Under the Curve (AUC), accuracy, precision, recall, and F1 score for predicting the TyG-index and AIP separately. The AUC, which reflects the comprehensive performance of the model across a spectrum of classification thresholds, serves as a pivotal metric in evaluating the model’s efficacy. A substantial AUC denotes an exceptional discernment capacity of the model in distinguishing between positive and negative samples. Accuracy, as the cornerstone metric for assessing classification prowess, represents the ratio of correctly predicted samples to the total samples. An increase in accuracy can somewhat indicate enhanced model performance, but accuracy is not a comprehensive metric when the sample distribution is not balanced. Precision, on the other hand, delineates the ratio of true positive samples to the model’s predicted positives within the entirety of predicted positive samples. Amplified precision signals a heightened precision in the identification of positive samples. Recall embodies the ratio of model-predicted positive samples to true positive samples, thus showcasing the model’s resilience in detecting all positive samples. The F1 score, embodying the harmonious fusion of precision and recall, acts as a comprehensive metric for gauging the holistic performance of the model. A higher F1 score corresponds to a superior overall model performance. The formulas for computing these metrics can be found in the supplementary materials (Supplementary Fig. S1).

### Machine learning algorithms

Regarding the machine learning algorithms, we adopted various models to conduct predictive analysis, aiming to explore the effectiveness of different models in addressing cardiovascular prediction problems. These models encompass Random Forest [28], Extra Trees [29], Bagging [30], Decision Tree [31], Extra Tree [29], XGBoost [32], LGBM [33], Gradient Boosting [34], Support Vector Machine (SVM) [35], AdaBoost [36], Label Propagation [37], Label Spreading [37], Linear SVM [35], Logistic Regression [38], Ridge Regression [39], Ridge CV [39], Multi-Layer Perceptron (MLP) [40], K Neighbors [41], Stochastic Gradient Descent Classifier (SGD) [42], Bernoulli NB [43], Perceptron [40], Passive Aggressive [44], Quadratic Discriminant Analysis [45], Gaussian NB [43], and Dummy. Through the utilization of these diverse machine learning models, we aim to evaluate their performance on the dataset comprehensively. This endeavor will aid in understanding which models exhibit optimal performance in handling our specific problem and what their strengths and weaknesses are. We conducted experiments using a five-fold cross-validation approach and employed multiple evaluation metrics to compare these models, including accuracy, precision, recall, and F1 score. These evaluation metrics will assist us in selecting the most suitable machine learning model for addressing our specific problem and provide guidance for future improvements and optimizations.

## Results

### Correlation analysis of data exploration

**Fig. 3:**
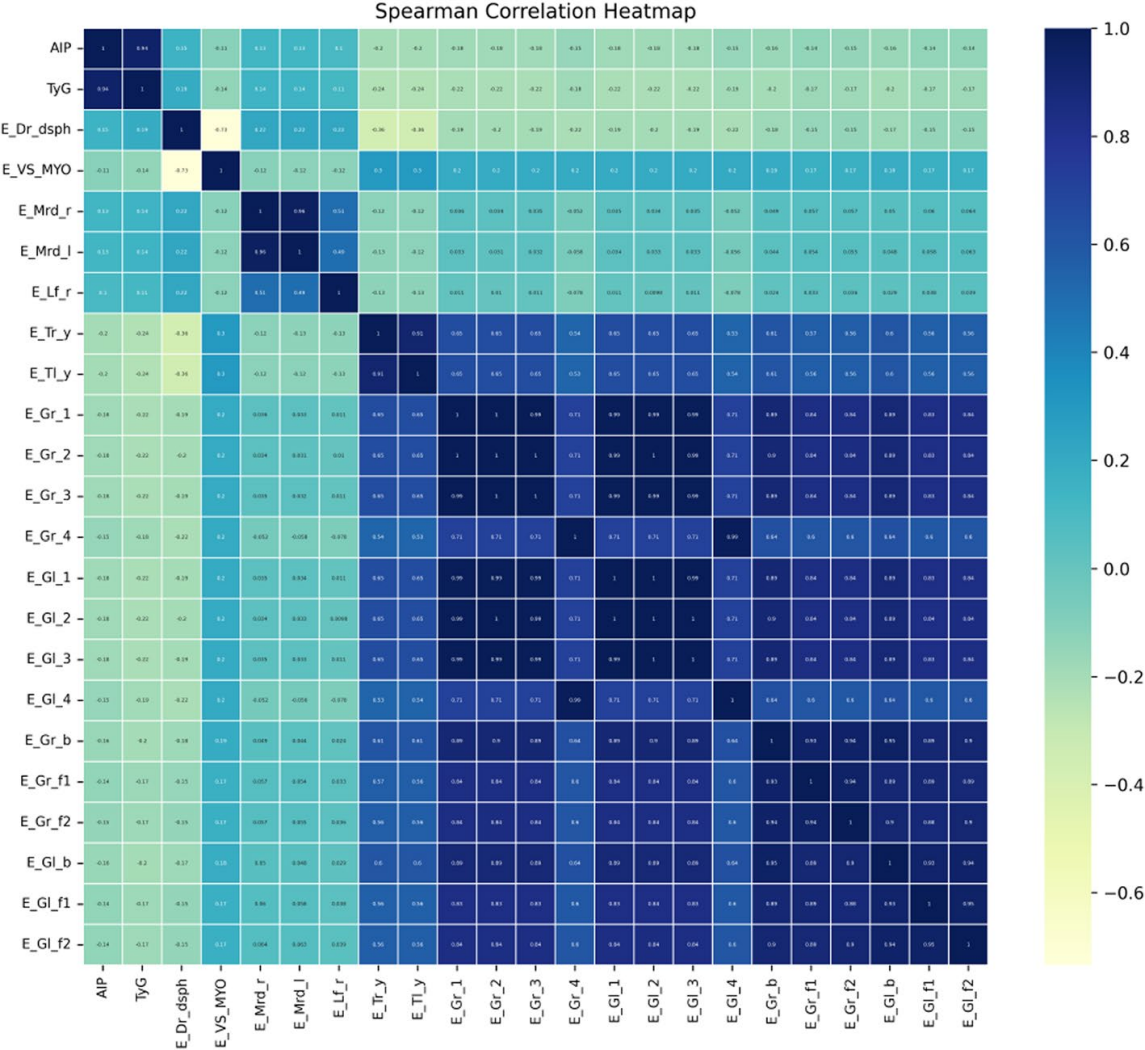
Heatmap of Spearman correlation ship in oculomics and clinic variables with TyG-index and AIP. Detailed descriptions of the input variables are shown in Supplementary Table S1. The standard names in the dictionary corresponding to different codes in the heatmap are shown in Supplementary Table S2. TyG-index, Triglyceride-glucose index. AIP, Atherogenic index of plasma

Initial processing of the raw dataset was conducted, as illustrated in Fig. 2 and Supplementary Fig. S3. After excluding variables with no significant statistical differences (*P*>0.001), we removed variables without clinical value and those directly calculable to yield outcome indicators. The remaining variables were used as input for our model. To illustrate the associations between the variables incorporated in the model, we generated a heatmap of Spearman coefficients (Fig. 3). Detailed descriptions of the input variables for the machine learning models are shown in Supplementary Table S1.

**Table 1:** Baseline Characteristics of Participants Stratified by Gender to develop a machine learning model to identify the TyG-index or AIP scales by clinical questionnaires and oculomics

Characteristics	Overall	Male	Female	*P* value^†^
N	32,122	14,254	17,868	
Age, years	45.02±19.30	44.22±19.70	45.65±18.95	<.001
Smoking status, n (%)				<.001
Current	8,397 (26.14%)	6,943 (48.71%)	1,454 (8.14%)	
Previous	3,502 (10.90%)	2,963 (20.79%)	539 (3.02%)	
Never	20,223 (62.96%)	4,348 (30.50%)	15,875 (88.85%)	
Smoking quantities, n (%)				<.001
Less than 5 boxes^a^	754 (2.35%)	404 (2.83%)	350 (1.96%)	
More than 5 boxes^b^	10,993 (34.22%)	9,408 (66.00%)	1,585 (8.87%)	
Never	20,375 (63.43%)	4,442 (31.16%)	15,933 (89.17%)	
Blood pressure				
SBP, mmHg	117.60±17.33	119.84±16.13	115.82±18.03	<.001
DBP, mmHg	75.06±11.07	77.29±10.52	73.28±10.35	<.001
BMI, kg/m2	23.24±3.56	23.49±3.45	23.04±3.64	<.001
BMI category, n (%)				<.001
<18.5 (Underweight)	1,985 (6.18%)	404 (2.83%)	1,235 (6.91%)	
18.5-24.9 (Normal)	20,110 (62.61%)	9,408 (66.00%)	11,454 (64.10%)	
$\ge$25 (Overweight or obesity)	10,027 (31.22%)	4,848 (34.01%)	5,179 (28.98%)	
Waist circumference, cm	79.61±10.75	82.35±10.39	77.42±10.52	<.001
Low HDL-C, n (%)	7,224 (22.49%)	4,226 (29.65%)	2,998 (16.78%)	<.001
Hypertriglyceridemia, n (%)	4,848 (15.09%)	2,643 (18.54%)	2,205 (12.34%)	<.001
Morbidities				
Hypertension, n (%)	21,912(68.21%)	8,956 (62.83%)	12,956 (72.51%)	<.001
Diabetes, n (%)	27,543 (85.74%)	11,900 (83.49%)	15,643 (87.55%)	<.001
MI, n (%)	3,986 (12.41%)	2,132 (14.96%)	1,854 (10.38%)	<.001
Stroke, n (%)	4,295 (13.37%)	2,270 (15.93%)	2,025 (11.33%)	<.001
Outcome				
TyG-index	8.51±0.67	8.64±0.70	8.41±0.63	<.001
AIP	0.34±0.32	0.41±0.33	0.28±0.30	<.001

Data were presented as mean±standard deviation, and proportions. AIP, Atherogenic index of plasma. BMI, Body mass index. DBP, Diastolic blood pressure. HDL-C, High density lipoprotein cholesterol. MI, Myocardial infarction. SBP, Systolic blood pressure. TyG-index, Triglyceride-glucose index.

^†^*P* value was derived from ANOVA or chi-square test. All statistical tests were performed in a two-sided manner with a significance level of *P* value <0.050.

^a^More than 5 boxes is defined from smoking entire life

^b^Less than 5 boxes is defined from smoking not the entire life

The findings are displayed in the form of a heatmap, where the color gradient from dark to light indicates the correlation from high to low. Due to space constraints, the variables in Fig. 3 are represented in the form of codes. Each code or character has a specific meaning, which can be found in the dictionaries of KNHANES for the years 2007-2009 and 2010-2012.

### Demographic analysis

In this study, a total of 32,122 participants (14,254 males and 17,868 females) from KNHANES were included in the final dataset. We gathered 36 input features comprising 11 demographic variables, 4 anthropometric parameters, and 21 ophthalmological measurements. These data were utilized to identify potential factors that may affect TyG-index or AIP, and the authentic calculated values of TyG-index or AIP were used as the outcome for constructing the machine learning model. Demographic characteristics of the participants are shown in Table 1. Baseline oculomics measurement specifics are detailed in Supplementary Tables S3 and S4 (Fig. 3).

**Fig. 4:**
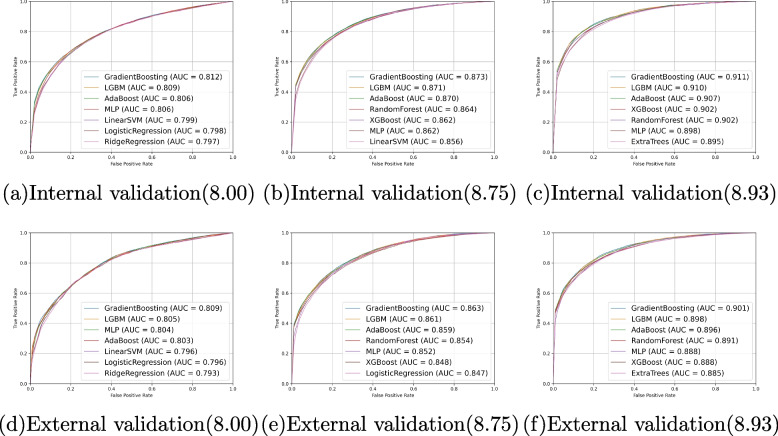
ROC and AUC of models use both oculomics and clinic variables as input variables. **a**-**c** The performance of our model using TyG-index=8.0, 8.75, or 8.93 as the cut-off point in the internal validation dataset is demonstrated. **d**-**f** The performance of our model using TyG-index=8.0, 8.75, or 8.93 as the cut-off point in the external validation dataset is demonstrated. AUC, Area under curve. TyG, Triglyceride-glucose index.ROC, Receiver operating characteristic

Our findings revealed that there were significant differences in the outcome values of TyG-index or AIP across all age and gender subgroups (*p*<0.001). Additionally, over 50% of the participants in the survey were either non-smokers (*n*=20223, 62.96%) or within the normal weight range (*n*=20110, 62.61%).

### Model performance and verification

We employed 25 machine learning algorithms for model establishment. After screening the correlation coefficient, the input variables consist of 36 variables, including 15 clinical indicators and 21 ophthalmic indicators.

**Fig. 5:**
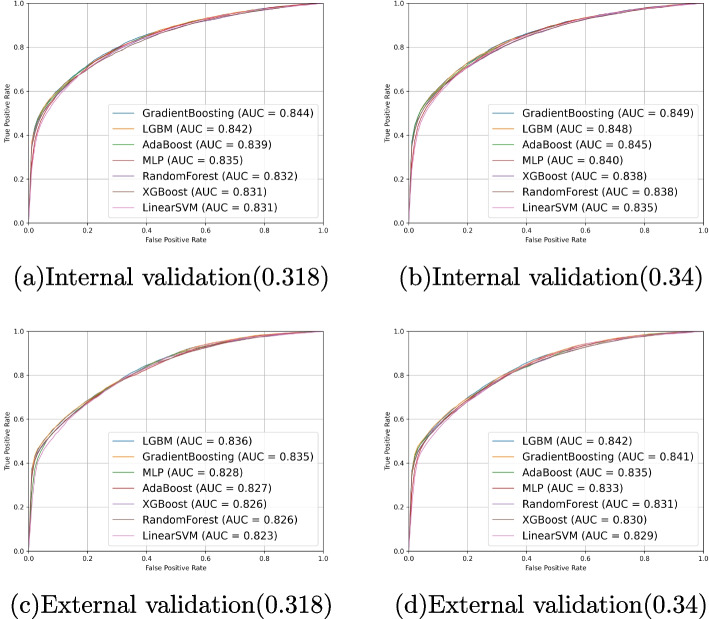
ROC and AUC of models use both oculomics and clinics as input variables. **a**-**b** The performance of our model using AIP=0.318 or 0.34 as the cut-off point in the internal validation dataset is demonstrated. **c**-**d** The performance of our model using AIP=0.318 or 0.34 as the cut-off point in the external validation dataset is demonstrated. AIP, Atherogenic index of plasma. AUC, Area under curve. ROC, Receiver operating characteristic

In terms of performance evaluation, based on the clinical questionnaire and oculomics measurement variables, we tested the overall model performance for five endpoints, yielding promising results (Tables 2 and 3). Moreover, due to the independent nature of the KNHANES database across different years, utilizing data from different years as internal and external validation sets is a common practice [46, 47]. In the predictive model employing all input factors, when the TyG-index cut-off values were 8.0, 8.75, and 8.93, the model’s AUC in the internal validation set was 0.812, 0.873, and 0.911, respectively, while in the external validation set, the AUC was 0.809, 0.863, and 0.901. We plot the model performance in the internal and external validation datasets with different TyG-index cut-off points as predicted outcomes in Fig. 4. As for the AIP endpoint, when set at 0.34, the internal and external validation sets are 0.849 and 0.842, respectively. Slightly poorer performance was observed for the 0.318 cut-off value, with AUCs of 0.844 and 0.836, but it remained reliable and compelling. The results of AIP endpoints are presented in Fig. 5.

**Fig. 6:**
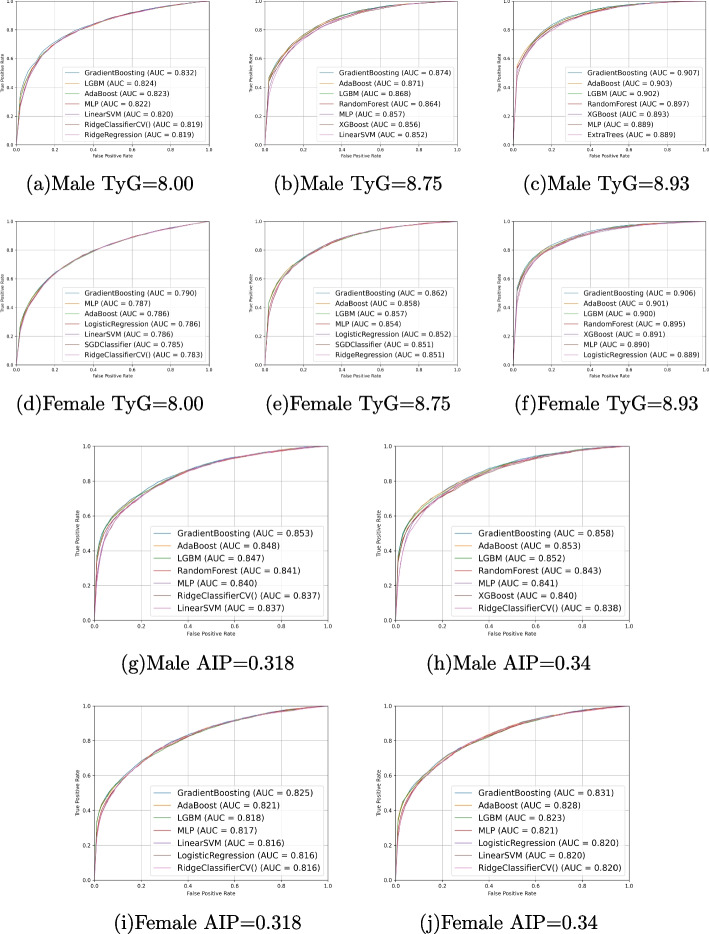
ROC and AUC in the internal validation models used both oculomics and clinic as input variables. **a**-**c**, **g**-**h** Five cut-off points of AIP and TyG-index in the male subgroup. **d**-**f**, **i**-**j** Five cut-off points of AIP and TyG-index in the female subgroup. AIP, Atherogenic index of plasma. AUC, Area under curve. ROC, Receiver operating characteristic. TyG-index, Triglyceride-glucose index

In addition, as gender is an independent risk factor for CVD [48], to eliminate the overfitting caused by gender bias in the overall predictive model, we conducted subgroup analyses for males and females separately. Results between gender subgroups are shown in Fig. 6. The AUC of models in male and female subgroups are most close only when the cut-off point of TyG-index is 8.93 (AUC=0.907 versus 0.906). The differences were observed when the cut-off point of TyG-index was set at 8, 8.9, and the cut-off point of AIP was set at 0.318, 0.34. The AUC values in males and females are corresponding with 0.832 and 0.790, 0.874 and 0.862, 0.853 and 0.825, 0.858 and 0.831.

**Table 2:** The model performance of 25 algorithms on the validation set and external validation set when TyG-index takes three different cut-off values

	TyG=8						TyG=8.75						TyG=8.93					
Model	Acc	AUC	F1	E-AUC	E-Acc	E-F1	Acc	AUC	F1	E-AUC	E-Acc	E-F1	Acc	AUC	F1	E-AUC	E-Acc	E-F1
GradientBoosting	0.789	0.812	0.870	0.809	0.808	0.884	0.820	0.873	0.694	0.863	0.804	0.666	0.874	0.911	0.709	0.901	0.865	0.693
LGBM	0.789	0.809	0.870	0.805	0.806	0.883	0.818	0.871	0.691	0.861	0.804	0.666	0.873	0.910	0.712	0.898	0.864	0.693
AdaBoost	0.785	0.806	0.868	0.803	0.803	0.881	0.816	0.870	0.689	0.859	0.805	0.671	0.872	0.907	0.703	0.896	0.863	0.685
RandomForest	0.784	0.796	0.867	0.791	0.802	0.881	0.813	0.864	0.683	0.854	0.800	0.661	0.870	0.902	0.693	0.891	0.862	0.676
XGBoost	0.781	0.796	0.865	0.793	0.800	0.878	0.812	0.862	0.686	0.848	0.795	0.658	0.867	0.902	0.701	0.888	0.858	0.679
MLP	0.786	0.806	0.868	0.804	0.805	0.882	0.806	0.862	0.679	0.852	0.794	0.654	0.861	0.898	0.684	0.888	0.856	0.671
RidgeCV	0.784	0.797	0.869	0.793	0.798	0.880	0.802	0.856	0.666	0.847	0.792	0.644	0.858	0.891	0.664	0.882	0.851	0.648
RidgeRegression	0.784	0.797	0.869	0.793	0.798	0.880	0.802	0.856	0.664	0.847	0.792	0.641	0.856	0.891	0.655	0.882	0.851	0.642
LogisticRegression	0.786	0.798	0.871	0.796	0.799	0.880	0.801	0.856	0.665	0.847	0.788	0.639	0.857	0.892	0.665	0.882	0.848	0.644
LinearSVM	0.785	0.799	0.870	0.796	0.798	0.879	0.802	0.856	0.666	0.847	0.793	0.645	0.858	0.892	0.669	0.883	0.851	0.652
ExtraTrees	0.783	0.788	0.867	0.787	0.800	0.879	0.807	0.854	0.678	0.846	0.796	0.658	0.863	0.895	0.680	0.885	0.857	0.665
SGD	0.781	0.794	0.869	0.791	0.797	0.880	0.798	0.854	0.644	0.844	0.783	0.617	0.853	0.891	0.655	0.880	0.844	0.636
PassiveAggressive	0.779	0.788	0.872	0.782	0.794	0.882	0.798	0.852	0.646	0.842	0.787	0.625	0.854	0.888	0.648	0.877	0.844	0.624
SVM	0.775	0.765	0.871	0.769	0.794	0.883	0.801	0.847	0.647	0.830	0.784	0.625	0.844	0.875	0.588	0.863	0.837	0.576
Perceptron	0.774	0.786	0.869	0.777	0.790	0.879	0.795	0.847	0.628	0.837	0.785	0.610	0.854	0.884	0.644	0.874	0.844	0.620
Bagging	0.758	0.753	0.845	0.748	0.768	0.853	0.798	0.836	0.656	0.822	0.782	0.633	0.860	0.874	0.678	0.863	0.854	0.669
GaussianNB	0.741	0.767	0.840	0.757	0.737	0.838	0.494	0.813	0.547	0.799	0.498	0.551	0.452	0.843	0.456	0.824	0.447	0.456
QuadraticDiscriminant	0.460	0.704	0.454	0.706	0.447	0.443	0.749	0.804	0.526	0.798	0.747	0.520	0.816	0.835	0.585	0.824	0.815	0.583
LabelPropagation	0.779	0.770	0.870	0.772	0.801	0.885	0.752	0.798	0.514	0.791	0.735	0.495	0.784	0.817	0.300	0.807	0.772	0.267
LabelSpreading	0.776	0.768	0.870	0.770	0.799	0.884	0.748	0.794	0.501	0.787	0.733	0.486	0.778	0.812	0.256	0.801	0.767	0.226
KNeighbors	0.760	0.718	0.849	0.725	0.773	0.860	0.757	0.782	0.585	0.775	0.747	0.576	0.819	0.811	0.563	0.799	0.809	0.541
DecisionTree	0.722	0.614	0.818	0.611	0.722	0.820	0.739	0.712	0.617	0.702	0.731	0.609	0.813	0.759	0.635	0.750	0.807	0.628
BernoulliNB	0.738	0.660	0.838	0.645	0.738	0.839	0.613	0.707	0.567	0.694	0.597	0.571	0.684	0.754	0.537	0.732	0.659	0.521
ExtraTree	0.709	0.597	0.810	0.605	0.722	0.821	0.726	0.693	0.591	0.687	0.719	0.588	0.797	0.730	0.594	0.717	0.785	0.581
Dummy	0.770	0.500	0.870	0.500	0.788	0.882	0.666	0.500	0.000	0.500	0.657	0.000	0.751	0.500	0.000	0.500	0.742	0.000

**Table 3:** The model performance of 25 algorithms on the validation set and external validation set when AIP takes two different cut-off values

	AIP=0.318						AIP=0.34					
Model	Acc	AUC	F1	E-AUC	E-Acc	E-F1	Acc	AUC	F1	E-AUC	E-Acc	E-F1
GradientBoosting	0.758	0.844	0.750	0.835	0.742	0.723	0.766	0.849	0.742	0.841	0.754	0.714
LGBM	0.757	0.842	0.746	0.836	0.744	0.724	0.766	0.848	0.739	0.842	0.754	0.713
AdaBoost	0.756	0.839	0.747	0.827	0.739	0.719	0.762	0.845	0.737	0.835	0.749	0.708
RandomForest	0.750	0.832	0.742	0.826	0.736	0.720	0.756	0.838	0.732	0.831	0.745	0.709
XGBoost	0.749	0.831	0.739	0.826	0.737	0.718	0.755	0.838	0.730	0.830	0.745	0.706
MLP	0.752	0.835	0.747	0.828	0.740	0.725	0.760	0.840	0.734	0.833	0.749	0.707
RidgeCV	0.751	0.831	0.746	0.823	0.736	0.720	0.756	0.835	0.734	0.829	0.745	0.709
RidgeRegression	0.751	0.830	0.746	0.823	0.735	0.720	0.755	0.835	0.734	0.828	0.744	0.709
LogisticRegression	0.751	0.830	0.744	0.823	0.734	0.716	0.756	0.835	0.734	0.828	0.746	0.709
LinearSVM	0.751	0.831	0.743	0.823	0.737	0.717	0.758	0.835	0.734	0.829	0.746	0.706
ExtraTrees	0.742	0.824	0.737	0.817	0.732	0.719	0.751	0.831	0.729	0.824	0.740	0.708
SGD	0.746	0.829	0.749	0.820	0.730	0.726	0.750	0.832	0.728	0.824	0.737	0.703
PassiveAggressive	0.744	0.822	0.734	0.812	0.731	0.706	0.750	0.828	0.725	0.820	0.739	0.700
SVM	0.745	0.827	0.726	0.820	0.735	0.709	0.749	0.830	0.707	0.825	0.742	0.685
Perceptron	0.737	0.813	0.729	0.799	0.719	0.703	0.752	0.826	0.726	0.818	0.740	0.698
Bagging	0.731	0.804	0.713	0.796	0.720	0.694	0.739	0.811	0.708	0.802	0.731	0.686
GaussianNB	0.675	0.789	0.723	0.778	0.661	0.708	0.666	0.793	0.710	0.783	0.652	0.694
QuadraticDiscriminant	0.652	0.786	0.533	0.783	0.660	0.522	0.661	0.790	0.520	0.786	0.670	0.512
LabelPropagation	0.687	0.759	0.693	0.758	0.685	0.696	0.690	0.764	0.680	0.762	0.686	0.678
LabelSpreading	0.683	0.756	0.691	0.755	0.682	0.695	0.686	0.761	0.679	0.759	0.682	0.678
KNeighbors	0.686	0.744	0.678	0.733	0.677	0.666	0.695	0.750	0.669	0.737	0.681	0.650
DecisionTree	0.675	0.675	0.680	0.669	0.669	0.669	0.685	0.685	0.675	0.674	0.675	0.6658
BernoulliNB	0.580	0.667	0.675	0.665	0.566	0.662	0.600	0.670	0.665	0.669	0.579	0.651
ExtraTree	0.669	0.669	0.674	0.655	0.655	0.652	0.674	0.673	0.664	0.663	0.663	0.645
Dummy	0.508	0.500	0.674	0.500	0.492	0.660	0.516	0.500	0.000	0.500	0.533	0.000

Regarding specific models, the Gradient Boosting method demonstrated the best performance across most experiments (Tables 2 and 3). The AUC of the LGBM method was only stronger than Gradient Boosting method with a slight advantage of 0.001 in the external validation set of 0.318 and 0.34. In the other three cut-off points, the LGBM method was slightly inferior to Gradient Boosting, although the results were similar. It is worth noting that out of the 25 methods employed, the top 7 methods were selected for illustration purposes (Tables 2 and 3, Supplementary Tables S5, S6, S7, and S8).

## Discussion

In this study, we realized the use of oculomics and clinic variables to predict the range of TyG-index and AIP by machine learning algorithms. The model demonstrated outstanding prediction efficiency with both internal and external datasets.

CVD is one of the most critical and dangerous chronic diseases in the world [49]. Early prevention and risk assessment of CVD are vital to patients or healthy adults. In order to achieve early prevention and risk assessment of CVD, many studies have been conducted on the risk factors of CVD. TyG-index and AIP combine traditional risk factors such as blood lipids and/or blood glucose that can reflect cardiovascular risk in different aspects. It has also been pointed out that TyG-index and AIP are better indicators of CVD than single biomarkers like TG and HDL-C [50, 51]. Recently, a systematic review documented that a greater TyG index might be individualistically linked to a greater incidence of atherosclerotic CVD among asymptomatic individuals. Several studies have confirmed the cross-sectional correlation between AIP and cardiovascular risk [52, 53]. Therefore, in primary screening of the population, especially in clinical practice, the TyG index and AIP can be considered to identify patients with high risk of CVD.

As previously reported, the eye provides a unique and transparent medium through which we can observe and measure various biological markers without any invasive procedures. Since oculomics was proposed in 2020, it has been widely discussed as a concept that combines AI, big data, and eye images [9]. Oculomics can be used for the prediction of many diseases, including sarcopenia and schizophrenia [46, 54, 55]. As for CVD, a review discussed the possibility of cardiovascular risk assessment based on oculomics [56, 57]. There are also studies combining oculomics and genomics to reveal aneurysm-related biomarkers [58]. In our study, we used oculomics and clinical features to predict cardiovascular risk. As for the cardiovascular risk, we selected different thresholds for TyG-index and AIP. The TyG-index cut-off point equals 8, and it is from a study involving 150,000 Koreans [21]. A graded positive association was observed between the TyG-index and CVD hospitalization. Per 1-unit increase in the TyG-index, a 16% increase in CVD hospitalization was demonstrated. When the cut-off point is the upper one-third of TyG values, we calculated 8.75 in our study population [22]. Compared with the lowest tertile of the TyG index, the highest tertile (tertile 3) was associated with a greater incidence of the composite outcome, myocardial infarction, stroke, and incident type 2 diabetes. When the cut-off point is the upper one-fourth of TyG values, we calculated 8.93 in our study population [23]. During 8.2 years of mean follow-up, the highest TyG index quartile demonstrated that these patients were at higher risk for stroke (HR=1.259; 95% CI 1.233-1.286), for MI (HR=1.313; 95% CI 1.28-1.346), and for both (HR=1.282; 95% CI 1.261-1.303) compared with participants in the lowest TyG index quartile.

As for the threshold of AIP, we chose 0.318 and 0.34. In type 2 diabetic subjects undergoing percutaneous coronary intervention, AIP plays an important role in predicting the prognosis. A recent study approved that when the value of AIP is higher than 0.318, the prognosis of the high AIP group was significantly worse than that of the low AIP group [20]. Another threshold for AIP was determined to be 0.34 in the study population [24]. High AIP ($\ge$0.34)presented the highest risk of cardiovascular deaths in patients with type 2 diabetes mellitus. Remarkably, in our study, the predictive efficacy was most conspicuous when TyG-index equated to 8.93, or AIP reached 0.318, suggesting that individuals demonstrating these particular indicators should be classified as the cohort at heightened risk for subsequent cardiovascular disease.

Gender differences in CVD have been discussed in recent studies [59]. A recent study showed that the absolute incidence of CVD in men was significantly higher than in women across all age groups [60]. Some researchers have observed that diabetes is more likely to be associated with ischemic heart disease in women than in men. And women are more likely to present with ischemia with no obstructive coronary arteries (INOCA) [61, 62]. On the treatment side, new treatments that are useful in men have not led to significant reductions in CVD mortality in women [63, 64]. So it is necessary to assess the gender-specific cardiovascular risks. In the subgroup analysis of this study, we found that the predictive efficacy of women was only stronger than that of men in both external validation sets when TyG=8.75 (AUC=0.849 and 0.867) and TyG=8.93 (0.881 and 0.908). Among all the remaining cut-off values of TyG and AIP, the AUC of the male subgroup was higher than that of the female subgroup. Our model has good predictive value for both men and women. Furthermore, this result suggests that the role of the TyG-index in predicting cardiovascular risk in women may be worthy of further exploration.

In this study, we used oculomics and non-invasive clinical data to predict the TyG index and AIP which are good indicators for CVD. Our research proposed that machine learning-based models successfully enhance the predictive performance for detecting abnormal ranges of AIP and TyG-index by using clinical questionnaires and oculomics. This conclusion holds promise for early identification of heightened cardiovascular risk by forecasting atypical TyG-index and AIP values in patients.

Therefore, our study has several strengths. First, we achieved good prediction of TYG and AIP by using variables from oculomics and clinical questionnaires. Both oculomics and clinical variables in the questionnaire were obtained non-invasively. These data are well recorded in the KNHANES database. We achieved the acquisition of these data and the construction of models at low cost. Low cost and non-invasiveness are important advantages for widespread application in clinical real-life scenarios [65]. Furthermore, although in its early stages, artificial intelligence (AI)-based oculomics methods may be a valuable non-invasive tool in primary care settings, enabling accurate diagnosis more cost-effectively and providing immediate results [66, 67]. Thus, our study has the advantage of enabling early detection and early intervention to prevent the progression of CVD in individuals at a low cost. In addition, due to the easy availability of ocular biomarkers, our study may also help eliminate barriers to uneven medical resources among regions with different economic levels.

## Limitation

Nevertheless, this study still bears several limitations. Firstly, whether the results of this study can be applied to populations of other races that differ significantly from Asians remains to be further validated. The KNHANES dataset is primarily composed of the East Asian population. Previous studies have indicated differences in upper eyelid anatomy between Asians and Caucasians [68]. Secondly, we only included a limited number of oculomics measurement variables due to data limitations. The original fundus photographs in the KNHANES database were inaccessible, preventing us from establishing an image-based predictive model. Incorporating imaging data such as oculomics photographs and retinal images could yield more high-throughput information and potentially improve the prediction of cardiovascular risk [10].

## Conclusion

In this study, we integrated two classifications of prognostic determinants, encompassing both clinical and oculomics textual parameters, in the formulation of the subject selection and model establishment. Throughout the experiment, we independently predicted the TyG-index and the AIP, yielding encouraging findings. These two variables collectively portray the metabolic status and may have an influence on the risk prediction of cardiovascular risks.

### Supplementary Information

**Supplementary Material 1.**

## Data Availability

No datasets were generated or analysed during the current study.

## Abbreviations

AIP: Atherogenic index of plasma
AUC: Area under the curve
BMI: Body mass index
CVD: Cardiovascular disease
DBP: Diastolic blood pressure
HDL-C: High density lipoprotein cholesterol
KNHANES: Korean National Health and Nutrition Examination Survey
MI: Myocardial infarction
SBP: Systolic blood pressure
TyG-index: Triglyceride-glucose index
