# Supplementary material for: Machine-learning-based models to predict cardiovascular risk using oculomics and clinic variables in KNHANES
Source: BioData Min. 2024 Apr 22;17:12. doi: 10.1186/s13040-024-00363-3 (PMC11034020; doi:10.1186/s13040-024-00363-3)
Supplement: Supplementary file 1 — Supplementary Material 1. [file 13040_2024_363_MOESM1_ESM.pdf]

$$Accuracy = \frac{TP + TN}{TP + FP + TN + FN}$$
$$Precision = \frac{TP}{TP + FP}$$
$$Recall = \frac{TP}{TP + FN}$$
$$F1\,Score = \frac{2 \times TP}{2 \times TP + FP + FN}$$

Supplementary Figure S1: Metrics for evaluation.
FN, False negative. FP, False positive. TN, True negative. TP, True positive.

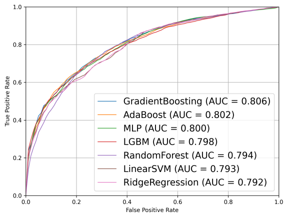

(a)Male TyG=8.00

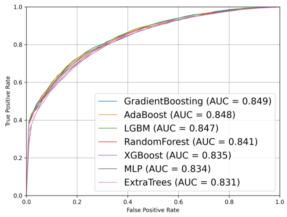

(b)Male TyG=8.75

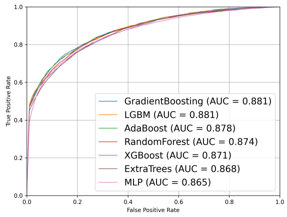

(c)Male TyG=8.93

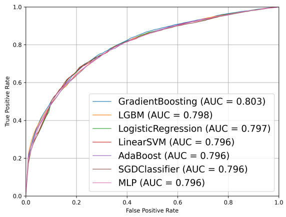

(d)Female TyG=8.00

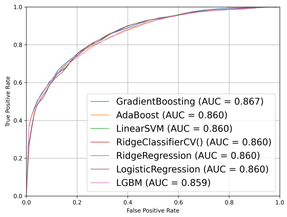

(e)Female TyG=8.75

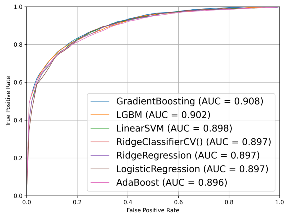

(f)Female TyG=8.93

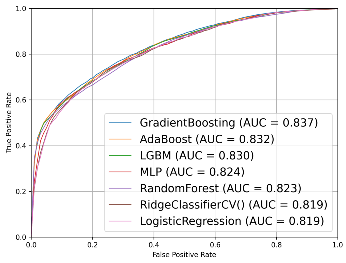

(g)Male AIP=0.318

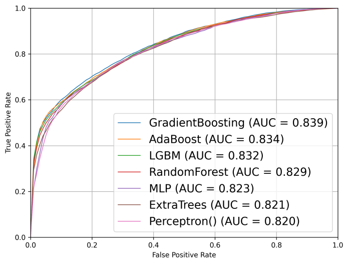

(h)Male AIP=0.34

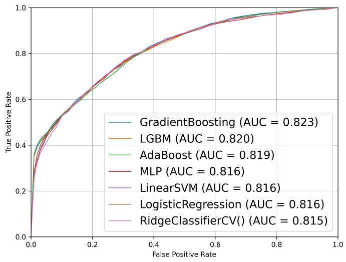

(i)Female AIP=0.318

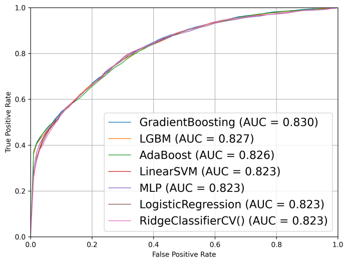

(j)Female AIP=0.34

Supplementary Figure S2: The performance of male and female in the external validation dataset when the input factors are combined with both oculomics and clinics. In this figure, 7 algorithms with the best performance in each model with AUC as the evaluation index are shown.

AIP, Atherogenic index of plasma. AUC, Area under the curve. TyG, Triglyceride-glucose.

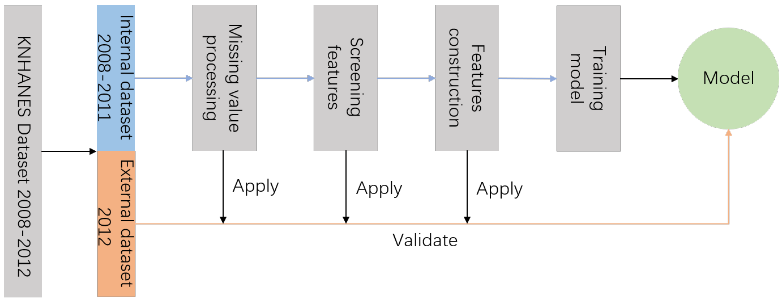

Supplementary Figure S3: Experimental flow chart.

Supplementary Table S1: Summary of the input variables for the TyG-index or AIP scales prediction model used in this study. Of particular note, within the oculomics variables, unless expressly stated otherwise, the term "both eyes" refers to this variable encompassing the two input variables of the left eye and the right eye. Otherwise, it should be interpreted by the specific instructions provided.

Category	Variable	Description	Unit or Class
Demographic variables	Sex		Male or female
	Age	Self-reported chronological age	Years
	Smoking status	Smoking status was defined based on self-reported cigarette use. The status was divided into three classes, including never smokers, past smokers, or current smokers, based on current smoking habits	Current smoker or not
	Smoking quantity	Smoke all life	<5 boxes, >5 boxes, or not
	Hypertension	Hypertension was defined as a previous diagnosis made by a physician	Yes or no
	Diabetes mellitus	Diabetes mellitus was defined as a previous diagnosis made by a physician	Yes or no
	Stroke	Stroke was defined as a previous diagnosis made by a physician	Yes or no
	Myocardial infarction	Myocardial infarction was defined as a previous diagnosis made by a physician	Yes or no
	High triglyceride	Hypertriglyceridemia was defined as a previous diagnosis made by a physician	Yes or no
	Low density lipoprotein cholesterol	Low density lipoprotein cholesterolemia was defined as a previous diagnosis made by a physician	Yes or no
	Obesity	Obesity was defined according to different BMI values, with BMI<18.5=low weght, BMI <18.5 ⩽ 25 = normal, and BMI ⩽ 25 = obesity.	Low weight, normal or obesity
Anthropometric parameters	Systolic blood pressure	Final systolic blood pressure (average of the second and third measurements)	mmHg
	Diastolic blood pressure	Final diastolic blood pressure (average of the second and third measurements)	mmHg
	Waist	Measured waist circumference value	Cm
	Body mass index	The participants measured their body weight and height barefoot and wearing light clothing. Body mass index is a person's weight (kg) divided by the square of height (m)	kg/m2
Ophthalmology examination	Myopia	Myopia was defined as a diagnosis made by an ophthalmologist	Yes or no
	Degree of myopia (right eye)	Automatic refraction of vision examination by Dsph	Degree
	Blepharoptosis (both eyes)	Marginal reflex distance 1 (MRD1※) is defined as the distance from the upper eyelid margin to the corneal light reflex in the primary position. We defined blepharoptosis as an MRD1 of <2 mm for either eye	>4mm, 3-3.9mm, 2-2.9mm, 1-1.9mm or <1mm
	Decreased levator function (right eye)	The levator muscle function test was also performed by measuring the upper eyelid excursion from downgaze to upgaze, excluding any influence of the frontalis muscle function, and sorted into normal (⩾ 12 mm) and decreased levator function (⩽ 11 mm) (Supplementary Figure 2)	>12mm, 8-11mm, 5-7mm or <4mm
	Cataract (both eyes)	The presence of cataract was determined clinically via slit-lamp examination by trained ophthalmologists. Lens Opacities Classification System III (LOCS III) photographic images were used as the reference to diagnose cataract	Yes, no, intraoculomics lens implantation, aphakia or <19 years old
	Glaucoma-corneal posterior deposit (both eyes)		Yes, no, <19 years old or not checked.
	Glaucoma-pseudoexfoliation syndrome (both eyes)		Yes, no, <19 years old or not checked.
	Glaucoma-iris atrophy or pupil sphincter paralysis (both eyes)		Yes, no, <19 years old or not checked.
	Glaucoma-Peripheral anterior chamber depth (both eyes)		<1/4, 1/4-1/2, >1/2 or <19 years old
	Glaucoma-mydriasis-optic disc hemorrhage (both eyes)		Yes, no, <19 years old or not checked.
	Glaucoma-mydriasis-optic nerve fiber layer defect-supratemporal (eyes)		Yes, no, <19 years old or not checked.
	Glaucoma-mydriasis-optic nerve fiber layer defect-infratemporal (eyes)		Yes, no, <19 years old or not checked.

※The definitions of blepharoptosis and MRD1 could be found on the eyewiki website. The measurement of MRD1 is the same as this article.

Supplementary Table S2: This table explains the standard names corresponding to different codes in the heatmap in the database dictionary.

Variable name	Description
HE_LHDL_st2	Low high density lipoprotein cholesterolemia
HE_hTG	Hypertriglyceridemia
sex	Sex
age	Age
DI3_lt	Stroke
DI5_lt	Myocardial infarction
BS3_1	Smoking right now
BS1_1	Smoking quantity
HE_HP	Hypertension
HE_DM	Diabetes
HE_sbp	Systolic blood pressure
HE_dbp	Diastolic blood pressure
HE_wc	Waist
HE_BMI	Body mass index
HE_obe	Obesity
E_Dr_dsph	Myopia degree of right eye
E_VS_MYO	Myopia disease
E_Mrd_r	Ptosis-right eye-mrd
E_Mrd_l	Ptosis-left eye-mrd
E_Lf_r	Ptosis-right eye-levator function
E_Tr_y	Cataract-right eye
E_Tl_y	Cataract-left eye
E_Gr_1	Glaucoma-right eye-keratic precipitates
E_Gr_2	Glaucoma-right eye-pseudoexfoliation syndrome
E_Gr_3	Glaucoma-right eye-iris atrophy or pupillary sphincter paralysis
E_Gr_4	Glaucoma-right eye-peripheral anterior chamber depth
E_Gl_1	Glaucoma-left eye-keratic precipitates
E_Gl_2	Glaucoma-left eye-pseudoexfoliation syndrome
E_Gl_3	Glaucoma-left eye-iris atrophy or pupillary sphincter paralysis
E_Gl_4	Glaucoma-left eye-peripheral anterior chamber depth
E_Gr_b	Glaucoma-right eye-no mydriasis-optic papilla hemorrhage
E_Gr_f1	Glaucoma-right eye-no mydriasis-retinal nerve fiber layer defect-superotemporal
E_Gr_f2	Glaucoma-right eye-no mydriasis-retinal nerve fiber layer defect-inferotemporal
E_Gl_b	Glaucoma-left eye-no mydriasis-optic papilla hemorrhage
E_Gl_f1	Glaucoma-left eye-no mydriasis-retinal nerve fiber layer defect-superotemporal
E_Gl_f2	Glaucoma-left eye-no mydriasis-retinal nerve fiber layer defect-inferotemporal

Supplementary Table S3: Baseline oculomics characteristics of the study participants stratified by gender.

Characteristics	Overall	Male (n=)	Female (n=)	P value
1 myopia disease, n (%)	16075 (50.04%)	7206 (50.55%)	8869 (49.64%)	0.102
2 ptosis-right eye-MRD, n (%)				<.001
⩾4mm	11698 (36.42%)	4703 (32.99%)	6995 (39.15%)	
3-3.9mm	10675 (33.23%)	4817 (33.79%)	5858 (32.78%)	
2-2.9mm	5971 (18.59%)	2881 (20.21%)	3090 (17.29%)	
1-1.9mm	2525 (7.86%)	1258 (8.83%)	1267 (7.09%)	
<1mm	769 (2.39%)	362 (2.54%)	407 (2.28%)	
not examine	484 (1.51%)	233 (1.63%)	251 (1.40%)	
3 ptosis-left eye-MRD, n (%)				<.001
⩾4mm	11628 (36.20%)	4668 (32.75%)	6960 (38.95%)	
3-3.9mm	10627 (33.08%)	4790 (33.60%)	5837 (32.67%)	
2-2.9mm	6032 (18.78%)	2897 (20.32%)	3135 (17.55%)	
1-1.9mm	2536 (7.89%)	1281 (8.99%)	1255 (7.02%)	
<1mm	808 (2.52%)	380 (2.67%)	428 (2.40%)	
not examine	491 (1.53%)	238 (1.67%)	253 (1.42%)	
4 ptosis-right eye-levator function, n (%)				0.009
⩾12mm	20787 (64.71%)	9147 (64.17%)	11640 (65.14%)	
8-11mm	9561 (29.76%)	4342 (30.46%)	5219 (29.21%)	
5-7mm	1144 (3.56%)	469 (3.29%)	675 (3.78%)	
<4mm	148 (0.46%)	64 (0.45%)	84 (0.47%)	
not examine	482 (1.50%)	232 (1.63%)	250 (1.40%)	
5 cataract-right eye, n (%)				<.001
yes	7730 (24.06%)	3436 (24.11%)	4294 (24.03%)	
No	18362 (57.16%)	7843 (55.02%)	10519 (58.87%)	
Intraocular lens eye	1445 (4.50%)	549 (3.85%)	896 (5.01%)	
aphakia	34 (0.11%)	20 (0.14%)	14 (0.08%)	
<19y	4057 (12.63%)	2166 (15.20%)	1891 (10.58%)	
not examine	494 (1.54%)	240 (1.68%)	254 (1.42%)	
6 cataract-left eye, n (%)				<.001
yes	7717 (24.02%)	3430 (24.06%)	4287 (23.99%)	
No	18364 (57.17%)	7846 (55.04%)	10518 (58.87%)	
Intraocular lens eye	1454 (4.53%)	554 (3.89%)	900 (5.04%)	
aphakia	27 (0.08%)	16 (0.11%)	11 (0.06%)	
<19y	4057 (12.63%)	2166 (15.20%)	1891 (10.58%)	
not examine	503 (1.57%)	242 (1.70%)	261 (1.46%)	
7 glaucoma-right eye-keratic precipitates, n (%)				<.001
yes	30 (0.09%)	15 (0.11%)	15 (0.08%)	
No	27422 (85.37%)	11789 (82.71%)	15633 (87.49%)	
<19y	4057 (12.63%)	2166 (15.20%)	1891 (10.58%)	
not examine	613 (1.91%)	284 (1.99%)	329 (1.84%)	
8 glaucoma-right eye-pseudoexfoliation syndrome, n (%)				<.001
yes	8 (0.02%)	2 (0.11%)	6 (0.03%)	
No	27448 (85.45%)	11803 (82.80%)	15645 (87.56%)	
<19y	4057 (12.63%)	2166 (15.20%)	1891 (10.58%)	
not examine	609 (1.90%)	283 (1.99%)	326 (1.82%)	
9 glaucoma-right eye-iris atrophy or pupillary sphincter paralysis, n (%)				<.001
yes	31 (0.10%)	12 (0.08%)	19 (0.11%)	
No	27411 (85.33%)	11787 (82.69%)	15624 (87.44%)	
<19y	4057 (12.63%)	2166 (15.20%)	1891 (10.58%)	
not examine	623 (1.94%)	289 (2.03%)	334 (1.87%)	

Supplementary Table S4: Continued of Supplementary Table S3

Characteristics	Overall	Male (n=)	Female (n=)	P value
10 glaucoma-right eye-peripheral anterior chamber depth, n (%)				<.001
<1/4	318 (0.99%)	94 (0.66%)	224 (1.25%)	
1/4-1/2	5393 (16.79%)	2150 (15.08%)	3243 (18.15%)	
>1/2	21598 (67.24%)	9506 (66.69%)	12092 (67.67%)	
<19y	4057 (12.63%)	2166 (15.20%)	1891 (10.58%)	
not examine	756 (2.35%)	338 (2.37%)	418 (2.34%)	
11 glaucoma-left eye-keratic precipitates, n (%)				<.001
yes	34 (0.11%)	13 (0.09%)	21 (0.12%)	
No	27424 (85.37%)	11792 (82.73%)	15632 (87.49%)	
<19y	4057 (12.63%)	2166 (15.20%)	1891 (10.58%)	
not examine	607 (1.89%)	283 (1.99%)	324 (1.81%)	
12 glaucoma-left eye-pseudoexfoliation syndrome, n (%)				<.001
yes	12 (0.04%)	3 (0.02%)	9 (0.05%)	
No	27447 (85.45%)	11802 (82.80%)	15645 (87.56%)	
<19y	4057 (12.63%)	2166 (15.20%)	1891 (10.58%)	
not examine	606 (1.89%)	283 (1.99%)	323 (1.81%)	
13 glaucoma-left eye-iris atrophy or pupillary sphincter paralysis, n (%)				<.001
yes	30 (0.09%)	12 (0.08%)	18 (0.10%)	
No	27428 (85.39%)	11791 (82.72%)	15637 (87.51%)	
<19y	4057 (12.63%)	2166 (15.20%)	1891 (10.58%)	
not examine	607 (1.89%)	285 (2.00%)	322 (1.80%)	
14 glaucoma-left eye-peripheral anterior chamber depth, n (%)				<.001
<1/4	309 (0.96%)	89 (0.62%)	220 (1.23%)	
1/4-1/2	5400 (16.81%)	2153 (15.10%)	3247 (18.17%)	
>1/2	21591 (67.22%)	9501 (66.65%)	12090 (67.66%)	
<19y	4057 (12.63%)	2166 (15.20%)	1891 (10.58%)	
not examine	765 (2.38%)	345 (2.42%)	420 (2.35%)	
15 glaucoma-right eye-no mydriasis-optic papilla hemorrhage, n (%)				<.001
yes	64 (0.20%)	29 (0.20%)	35 (0.20%)	
No	27139 (84.49%)	11672 (81.89%)	15467 (86.56%)	
<19y	4057 (12.63%)	2166 (15.20%)	1891 (10.58%)	
not examine	862 (2.68%)	387 (2.72%)	475 (2.66%)	
16 glaucoma-right eye-no mydriasis-retinal nerve fiber layer defect-superotemporal, n (%)				<.001
yes	617 (1.92%)	319 (2.24%)	298 (1.67%)	
No	26475 (82.42%)	11327 (79.47%)	15148 (84.78%)	
<19y	4057 (12.63%)	2166 (15.20%)	1891 (10.58%)	
not examine	973 (3.03%)	442 (3.10%)	531 (2.97%)	
17 glaucoma-right eye-no mydriasis-retinal nerve fiber layer defect-inferotemporal, n (%)				<.001
yes	462 (1.44%)	237 (1.66%)	225 (1.26%)	
No	26584 (82.76%)	11388 (79.89%)	15196 (85.05%)	
<19y	4057 (12.63%)	2166 (15.20%)	1891 (10.58%)	
not examine	1019 (3.17%)	463 (3.25%)	556 (3.11%)	
18 glaucoma-left eye-no mydriasis-optic papilla hemorrhage, n (%)				<.001
yes	67 (0.21%)	27 (0.19%)	40 (0.22%)	
No	27093 (84.34%)	11644 (81.69%)	15449 (86.46%)	
<19y	4057 (12.63%)	2166 (15.20%)	1891 (10.58%)	
not examine	905 (2.82%)	417 (2.93%)	488 (2.73%)	
19 glaucoma- left eye-no mydriasis-retinal nerve fiber layer defect-superotemporal, n (%)				<.001
yes	641 (2.00%)	343 (2.41%)	298 (1.67%)	
No	26395 (82.17%)	11261 (79.00%)	15134 (84.70%)	
<19y	4057 (12.63%)	2166 (15.20%)	1891 (10.58%)	
not examine	1029 (3.20%)	484 (3.40%)	545 (3.05%)	
20 glaucoma- left eye-no mydriasis-retinal nerve fiber layer defect-inferotemporal, n (%)				<.001
yes	471 (1.47%)	249 (1.75%)	222 (1.24%)	
No	26570 (82.72%)	11357 (79.68%)	15213 (85.14%)	
<19y	4057 (12.63%)	2166 (15.20%)	1891 (10.58%)	
not examine	1024 (3.19%)	482 (3.38%)	542 (3.03%)	

P value was derived from chi-square test. Data were presented as proportions. All statistical tests were performed in a two-sided manner with a significance level of P value<0.050.

Supplementary Table S5: The model performance of 25 algorithms on the validation set and external validation set in the male subgroup when TyG-index takes three different cut-off values.

	TyG=8.0						TyG=8.75						TyG=8.93					
Model	Acc	AUC	F1	E-AUC	E-Acc	E-F1	Acc	AUC	F1	E-AUC	E-Acc	E-F1	Acc	AUC	F1	E-AUC	E-Acc	E-F1
GradientBoosting	0.833	0.832	0.904	0.806	0.841	0.909	0.794	0.874	0.725	0.849	0.775	0.693	0.846	0.907	0.730	0.881	0.834	0.694
AdaBoost	0.829	0.823	0.900	0.802	0.836	0.906	0.795	0.871	0.729	0.848	0.776	0.699	0.848	0.903	0.735	0.878	0.836	0.702
LGBM	0.830	0.824	0.902	0.798	0.833	0.904	0.791	0.868	0.722	0.847	0.771	0.689	0.840	0.902	0.723	0.881	0.835	0.703
XGBOOST	0.823	0.809	0.897	0.784	0.826	0.899	0.781	0.856	0.712	0.835	0.762	0.681	0.835	0.893	0.720	0.871	0.828	0.694
RandomForest	0.830	0.815	0.902	0.794	0.836	0.906	0.789	0.864	0.719	0.841	0.769	0.684	0.840	0.897	0.714	0.874	0.831	0.682
MLP	0.831	0.822	0.902	0.800	0.842	0.909	0.781	0.857	0.711	0.834	0.764	0.678	0.834	0.889	0.707	0.865	0.826	0.678
RidgeCV	0.831	0.819	0.903	0.792	0.843	0.910	0.777	0.852	0.706	0.829	0.762	0.669	0.827	0.879	0.692	0.858	0.819	0.659
RidgeRegression	0.831	0.819	0.903	0.792	0.843	0.910	0.777	0.852	0.706	0.829	0.763	0.670	0.827	0.879	0.694	0.858	0.820	0.664
LinearSVM	0.834	0.820	0.905	0.793	0.841	0.909	0.778	0.852	0.707	0.829	0.765	0.674	0.828	0.881	0.696	0.861	0.822	0.668
LogisticRegression	0.834	0.818	0.905	0.789	0.838	0.908	0.778	0.851	0.705	0.829	0.763	0.672	0.829	0.880	0.692	0.860	0.821	0.662
SGD	0.829	0.815	0.901	0.787	0.836	0.909	0.773	0.850	0.699	0.827	0.758	0.665	0.825	0.881	0.682	0.859	0.818	0.653
PassiveAggressive	0.828	0.804	0.904	0.778	0.838	0.910	0.776	0.847	0.698	0.824	0.756	0.655	0.829	0.879	0.697	0.861	0.820	0.667
Perceptron	0.828	0.807	0.904	0.780	0.841	0.912	0.776	0.845	0.698	0.820	0.754	0.656	0.828	0.877	0.687	0.854	0.817	0.652
ExtraTrees	0.829	0.805	0.901	0.790	0.835	0.905	0.777	0.851	0.706	0.831	0.759	0.674	0.832	0.889	0.701	0.868	0.824	0.672
SVM	0.831	0.753	0.903	0.753	0.846	0.913	0.778	0.846	0.693	0.814	0.754	0.645	0.810	0.866	0.615	0.843	0.803	0.580
Bagging	0.807	0.772	0.884	0.745	0.804	0.883	0.767	0.839	0.686	0.816	0.753	0.660	0.826	0.872	0.694	0.850	0.819	0.669
GaussianNB	0.794	0.792	0.875	0.759	0.777	0.864	0.539	0.808	0.618	0.780	0.531	0.607	0.470	0.833	0.527	0.802	0.459	0.509
LabelPropagation	0.829	0.788	0.903	0.767	0.845	0.912	0.724	0.792	0.613	0.801	0.725	0.613	0.742	0.818	0.379	0.824	0.746	0.372
LabelSpreading	0.829	0.786	0.903	0.765	0.845	0.913	0.719	0.789	0.601	0.799	0.719	0.601	0.730	0.814	0.323	0.820	0.733	0.311
Kneighbors	0.812	0.723	0.890	0.716	0.816	0.893	0.724	0.777	0.634	0.764	0.717	0.622	0.783	0.808	0.605	0.800	0.783	0.592
QuadraticDiscriminantAnalysis	0.822	0.500	0.902	0.500	0.840	0.913	0.703	0.797	0.645	0.780	0.694	0.627	0.737	0.825	0.658	0.809	0.725	0.635
BernoulliNB	0.791	0.685	0.873	0.655	0.780	0.866	0.534	0.715	0.612	0.702	0.527	0.604	0.478	0.753	0.523	0.738	0.473	0.510
DecisionTree	0.762	0.616	0.853	0.610	0.766	0.857	0.723	0.716	0.665	0.694	0.705	0.638	0.774	0.745	0.653	0.735	0.769	0.638
ExtraTree	0.763	0.606	0.855	0.600	0.765	0.858	0.703	0.692	0.636	0.673	0.687	0.610	0.760	0.724	0.625	0.714	0.754	0.609
Dummy	0.822	0.500	0.902	0.500	0.840	0.913	0.592	0.500	0.000	0.500	0.586	0.000	0.681	0.500	0.000	0.500	0.682	0.000

Supplementary Table S6: The model performance of 25 algorithms on the validation set and external validation set in the male subgroup when AIP takes two different cut-off values.

	AIP=0.318						AIP=0.34					
Model	Acc	AUC	F1	E-AUC	E-Acc	E-F1	Acc	AUC	F1	E-AUC	E-Acc	E-F1
GradientBoosting	0.768	0.853	0.805	0.837	0.746	0.778	0.768	0.858	0.796	0.839	0.746	0.764
AdaBoost	0.759	0.848	0.799	0.832	0.740	0.772	0.766	0.853	0.794	0.834	0.740	0.759
LGBM	0.761	0.847	0.798	0.830	0.736	0.767	0.760	0.852	0.788	0.832	0.737	0.754
XGBOOST	0.745	0.836	0.785	0.816	0.723	0.756	0.749	0.840	0.779	0.819	0.728	0.748
RandomForest	0.756	0.841	0.796	0.823	0.732	0.768	0.756	0.843	0.786	0.829	0.736	0.757
MLP	0.756	0.840	0.791	0.824	0.736	0.763	0.759	0.841	0.792	0.823	0.739	0.764
RidgeCV	0.757	0.837	0.800	0.819	0.735	0.773	0.760	0.838	0.792	0.820	0.737	0.762
RidgeRegression	0.756	0.836	0.800	0.819	0.734	0.774	0.757	0.838	0.790	0.819	0.735	0.762
LinearSVM	0.757	0.837	0.798	0.819	0.737	0.772	0.760	0.838	0.791	0.820	0.738	0.761
LogisticRegression	0.756	0.836	0.801	0.819	0.736	0.778	0.757	0.836	0.793	0.818	0.735	0.767
SGD	0.743	0.834	0.799	0.816	0.723	0.777	0.754	0.836	0.791	0.818	0.731	0.765
PassiveAggressive	0.752	0.831	0.796	0.814	0.733	0.772	0.750	0.832	0.788	0.813	0.729	0.761
Perceptron	0.741	0.828	0.796	0.813	0.725	0.776	0.748	0.832	0.789	0.820	0.734	0.770
ExtraTrees	0.746	0.828	0.790	0.815	0.729	0.769	0.746	0.834	0.778	0.821	0.738	0.763
SVM	0.744	0.825	0.788	0.810	0.727	0.773	0.742	0.824	0.777	0.807	0.725	0.760
Bagging	0.732	0.813	0.766	0.798	0.714	0.741	0.735	0.819	0.759	0.799	0.716	0.729
GaussianNB	0.664	0.797	0.762	0.772	0.645	0.743	0.648	0.799	0.746	0.778	0.628	0.724
LabelPropagation	0.695	0.773	0.777	0.765	0.679	0.766	0.685	0.767	0.763	0.757	0.676	0.757
LabelSpreading	0.691	0.770	0.776	0.763	0.674	0.765	0.681	0.764	0.763	0.755	0.670	0.755
Kneighbors	0.701	0.753	0.749	0.725	0.679	0.727	0.693	0.743	0.731	0.724	0.675	0.712
QuadraticDiscriminantAnalysis	0.683	0.724	0.726	0.694	0.659	0.692	0.672	0.728	0.688	0.707	0.647	0.648
BernoulliNB	0.658	0.681	0.757	0.682	0.640	0.739	0.643	0.680	0.741	0.677	0.623	0.721
DecisionTree	0.689	0.676	0.741	0.664	0.672	0.717	0.691	0.685	0.731	0.671	0.674	0.707
ExtraTree	0.679	0.667	0.732	0.658	0.665	0.711	0.676	0.668	0.719	0.657	0.662	0.697
Dummy	0.599	0.500	0.749	0.500	0.584	0.738	0.577	0.500	0.731	0.500	0.561	0.719

Supplementary Table S7: The model performance of 25 algorithms on the validation set and external validation set in the female subgroup when TyG-index takes three different cut-off values.

	TyG=8.0						TyG=8.75						TyG=8.93					
Model	Acc	AUC	F1	E-AUC	E-Acc	E-F1	Acc	AUC	F1	E-AUC	E-Acc	E-F1	Acc	AUC	F1	E-AUC	E-Acc	E-F1
GradientBoosting	0.755	0.790	0.841	0.803	0.781	0.861	0.838	0.862	0.650	0.867	0.825	0.636	0.895	0.906	0.677	0.908	0.888	0.681
AdaBoost	0.755	0.786	0.839	0.796	0.777	0.858	0.837	0.858	0.650	0.860	0.824	0.637	0.893	0.901	0.669	0.896	0.887	0.674
MLP	0.757	0.787	0.841	0.796	0.776	0.857	0.826	0.854	0.629	0.856	0.821	0.632	0.881	0.890	0.635	0.889	0.878	0.646
LGBM	0.752	0.783	0.838	0.798	0.777	0.858	0.836	0.857	0.646	0.859	0.823	0.634	0.891	0.900	0.668	0.902	0.885	0.674
LinearSVM	0.756	0.786	0.843	0.796	0.778	0.860	0.824	0.851	0.622	0.860	0.819	0.623	0.883	0.889	0.638	0.898	0.879	0.646
LogisticRegression	0.757	0.786	0.844	0.797	0.777	0.860	0.824	0.852	0.622	0.860	0.816	0.617	0.877	0.889	0.611	0.897	0.870	0.609
RidgeCV	0.756	0.783	0.843	0.794	0.774	0.857	0.824	0.851	0.621	0.860	0.819	0.624	0.882	0.888	0.635	0.897	0.878	0.647
RidgeRegression	0.756	0.783	0.843	0.794	0.774	0.857	0.824	0.851	0.621	0.860	0.819	0.623	0.883	0.888	0.635	0.897	0.878	0.647
SGD	0.754	0.785	0.840	0.796	0.773	0.856	0.816	0.851	0.583	0.859	0.811	0.585	0.873	0.889	0.611	0.896	0.869	0.617
PassiveAggressive	0.747	0.778	0.844	0.789	0.768	0.860	0.820	0.848	0.600	0.855	0.816	0.605	0.878	0.887	0.615	0.893	0.871	0.620
RandomForest	0.744	0.773	0.834	0.787	0.777	0.859	0.834	0.851	0.638	0.856	0.823	0.631	0.890	0.895	0.655	0.896	0.884	0.661
SVM	0.744	0.769	0.845	0.784	0.769	0.863	0.820	0.838	0.581	0.840	0.813	0.585	0.872	0.857	0.556	0.869	0.867	0.570
XGBoost	0.743	0.769	0.831	0.781	0.770	0.853	0.829	0.846	0.636	0.844	0.817	0.628	0.888	0.891	0.666	0.892	0.880	0.665
Perceptron	0.726	0.777	0.838	0.782	0.747	0.852	0.820	0.843	0.578	0.849	0.813	0.571	0.879	0.883	0.607	0.890	0.875	0.619
ExtraTrees	0.742	0.765	0.832	0.779	0.769	0.853	0.824	0.841	0.622	0.846	0.817	0.623	0.881	0.885	0.625	0.885	0.878	0.644
LabelPropagation	0.748	0.767	0.843	0.782	0.771	0.860	0.794	0.824	0.448	0.834	0.782	0.442	0.822	0.829	0.202	0.826	0.801	0.151
LabelSpreading	0.746	0.767	0.844	0.782	0.770	0.861	0.792	0.823	0.436	0.832	0.780	0.430	0.819	0.827	0.172	0.821	0.799	0.130
Bagging	0.719	0.730	0.808	0.740	0.737	0.824	0.822	0.820	0.617	0.823	0.809	0.610	0.883	0.866	0.642	0.862	0.879	0.659
GaussianNB	0.724	0.750	0.805	0.756	0.743	0.826	0.585	0.810	0.537	0.815	0.583	0.552	0.493	0.841	0.410	0.837	0.489	0.429
QuadraticDiscriminantAnalysis	0.728	0.500	0.843	0.500	0.748	0.856	0.776	0.798	0.479	0.807	0.778	0.492	0.836	0.834	0.525	0.832	0.830	0.522
Kneighbors	0.722	0.708	0.816	0.729	0.742	0.832	0.797	0.785	0.573	0.799	0.799	0.590	0.848	0.799	0.497	0.800	0.833	0.473
DecisionTree	0.679	0.603	0.777	0.609	0.692	0.790	0.759	0.704	0.569	0.701	0.750	0.574	0.840	0.753	0.596	0.745	0.829	0.598
ExtraTree	0.675	0.594	0.775	0.606	0.693	0.792	0.745	0.680	0.535	0.689	0.745	0.558	0.810	0.694	0.507	0.693	0.805	0.519
BernoulliNB	0.695	0.647	0.809	0.646	0.704	0.816	0.695	0.690	0.496	0.680	0.672	0.512	0.726	0.659	0.366	0.619	0.672	0.352
Dummy	0.728	0.500	0.843	0.500	0.748	0.856	0.726	0.500	0.000	0.500	0.711	0.000	0.807	0.500	0.000	0.500	0.788	0.000

Supplementary Table S8: The model performance of 25 algorithms on the validation set and external validation set in the female subgroup when AIP takes two different cut-off values.

	AIP=0.318						AIP=0.34					
Model	Acc	AUC	F1	E-AUC	E-Acc	E-F1	Acc	AUC	F1	E-AUC	E-Acc	E-F1
GradientBoosting	0.752	0.825	0.682	0.823	0.743	0.665	0.766	0.831	0.673	0.830	0.757	0.647
AdaBoost	0.748	0.821	0.680	0.819	0.737	0.659	0.764	0.828	0.673	0.826	0.754	0.647
MLP	0.746	0.817	0.672	0.816	0.740	0.652	0.758	0.821	0.657	0.823	0.756	0.638
LGBM	0.749	0.818	0.678	0.820	0.741	0.660	0.760	0.823	0.666	0.827	0.757	0.648
LinearSVM	0.749	0.816	0.680	0.816	0.741	0.662	0.759	0.820	0.668	0.823	0.754	0.648
LogisticRegression	0.749	0.816	0.680	0.816	0.740	0.662	0.758	0.820	0.668	0.823	0.755	0.651
RidgeCV	0.749	0.816	0.684	0.815	0.741	0.668	0.757	0.820	0.670	0.823	0.754	0.655
RidgeRegression	0.749	0.816	0.684	0.815	0.741	0.668	0.757	0.820	0.670	0.823	0.754	0.655
SGD	0.746	0.814	0.679	0.814	0.739	0.663	0.746	0.819	0.678	0.821	0.741	0.666
PassiveAggressive	0.745	0.810	0.669	0.810	0.737	0.653	0.758	0.817	0.657	0.818	0.750	0.631
RandomForest	0.741	0.809	0.674	0.811	0.740	0.670	0.757	0.814	0.667	0.817	0.751	0.652
SVM	0.742	0.805	0.640	0.805	0.739	0.615	0.750	0.798	0.613	0.806	0.752	0.583
XGBoost	0.739	0.804	0.670	0.805	0.734	0.657	0.751	0.809	0.661	0.812	0.748	0.645
Perceptron	0.738	0.800	0.642	0.799	0.732	0.616	0.755	0.813	0.644	0.813	0.749	0.620
ExtraTrees	0.734	0.800	0.665	0.801	0.729	0.656	0.750	0.804	0.663	0.807	0.745	0.645
LabelPropagation	0.711	0.788	0.591	0.792	0.712	0.597	0.723	0.793	0.565	0.800	0.725	0.569
LabelSpreading	0.709	0.786	0.589	0.790	0.708	0.595	0.721	0.791	0.563	0.798	0.721	0.565
Bagging	0.729	0.780	0.653	0.778	0.720	0.634	0.739	0.786	0.638	0.783	0.733	0.622
GaussianNB	0.679	0.777	0.684	0.778	0.668	0.674	0.674	0.781	0.669	0.785	0.666	0.661
QuadraticDiscriminantAnalysis	0.698	0.770	0.540	0.776	0.710	0.531	0.721	0.775	0.567	0.780	0.735	0.564
Kneighbors	0.710	0.759	0.644	0.759	0.711	0.637	0.727	0.764	0.639	0.766	0.727	0.630
DecisionTree	0.673	0.668	0.626	0.660	0.663	0.614	0.677	0.668	0.611	0.669	0.677	0.606
ExtraTree	0.657	0.652	0.609	0.653	0.657	0.604	0.667	0.657	0.597	0.647	0.657	0.580
BernoulliNB	0.592	0.650	0.586	0.651	0.552	0.578	0.595	0.653	0.565	0.654	0.561	0.562
Dummy	0.565	0.500	0.000	0.500	0.580	0.000	0.590	0.500	0.000	0.500	0.606	0.000

Supplementary Table S9: This table describes the number of people at high risk for cardiovascular disease at each cut-off point. Here we define values exceeding cut-off values as positive.

	AIP		TYG		
Thresholds	0.318	0.34	8	8.75	8.93
Positivity rate	50.50%	48.10%	77.30%	33.60%	25.10%
Negative rate	49.50%	51.90%	22.70%	66.40%	74.90%
